# Supplementary material for: Patterns of Intron Gain and Loss in Fungi
Source: PLoS Biol. 2004 Nov 30;2(12):e422. doi: 10.1371/journal.pbio.0020422 (PMC532390; doi:10.1371/journal.pbio.0020422)
Supplement: Table S1 — Also available at http://genes.mit.edu/NielsenEtAl/. (4.3 MB ZIP). [file pbio.0020422.st001.zip › NielsenEtAl/html/588.html]

AN4033.1.NCU09249.1.MG01652.1.FG07088.1


```
 CLUSTAL W (1.82) Multiple Sequence Alignments - Introns Inserted


Sequence 1: MG01652.1	145 aa
Sequence 2: FG07088.1	120 aa
Sequence 3: NCU09249.1	117 aa
Sequence 4: AN4033.1	123 aa
Alignment Length: 154 aa
Number Identitical Residues: 63 aa
Alignment Score (without introns) 2992


MG01652.1 	---MASQ-QVSVKKQQE1LQAQYSTYKDTLQQVARKIGDVEQEAEEH-~----K~SVFAI
NCU09249.1	---MAQQ-PAAAKKQQD1LQLQYTTYKNTLQQIAQKIGDVEQEAEEH-~----K2LVLET
FG07088.1 	MSTAAAG-QVPAKKQQD1LQVTYSNYKNTLQQIAQRIGDIEQEAEEH-~----K2LVLET
AN4033.1  	---MASQPQIAPKKQQE1LQAQYTNFKNTLQQLAQKIGDIEQEAEEHN2CALRR~LVIDT
          	    *  .  . ****: **  *:.:*:****:*::***:*******. .:  :  *:  

MG01652.1 	SSSCNSPLKVAIWRAGQAVKTPAAVTHSLLMVLAN1RKCFRLINGVLVERTVKDVVPALK
NCU09249.1	LDPLPA----------------------------D~RKCFRMINGVLVERTVEDVIPALK
FG07088.1 	LEPLSE----------------------------D~RKCFRLINGVLVERTVKDVVPALK
AN4033.1  	LEPLPQ----------------------------D~RTCFRMVNGVLVERTVADVLPTLK
          	 ..                               : *.***::********* **:*:**

MG01652.1 	TNAEGLRKVLEDLVKQYKGKEEEFEKWK0KKNNVQVVQS
NCU09249.1	TNAEGLKKVLNDLVKQYQNKQEELDKWK0KKNNVQVVQS
FG07088.1 	TNQEGLRKVLEDLVKQYKTKQDDLEKWK0KKHNVQVVQQ
AN4033.1  	TNSDGLKQVLEDMLKQYKSKQSELDNWK0KKNNIQVVQP
          	** :**::**:*::***: *:.::::** **:*:****
```
